# Automated ITSS extraction combined with R2* values obtained from enhanced T2*-weighted angiography in magnetic resonance imaging: a promising approach for differentiate cervical adenocarcinoma from squamous carcinoma

**DOI:** 10.3389/fonc.2025.1578525

**Published:** 2025-11-18

**Authors:** Anliang Chen, Xing Meng, Changjun Ma, Shifeng Tian, Qingwei Song, Ailian Liu, Mingrui Zhuang, Hongkai Wang

**Affiliations:** 1 Department of Radiology, The First Affiliated Hospital of Dalian Medical University, Dalian, Liaoning, China; 2 Dalian Medical Imaging Artificial Intelligence Engineering Technology Research Center, Dalian, Liaoning, China; 3 Department of Radiology, Dalian Women and Children’s Medical Group, Dalian, Liaoning, China; 4 Technology Innovation Center of Hyperpolarized MRI, Dalian, Liaoning, China; 5 School of Biomedical Engineering, Faculty of Medicine, Dalian University of Technology, Dalian, Liaoning, China; 6 Liaoning Key Laboratory of Integrated Circuit and Biomedical Electronic System, Dalian, Liaoning, China

**Keywords:** cervical adenocarcinoma, cervical squamous carcinoma, intertumoral susceptibility signal, R2* value, ESWAN

## Abstract

**Purpose:**

This study aims to evaluate the efficacy of utilizing automated intertumoral susceptibility signal (ITSS) intensity extraction combined with R2* values derived from enhanced T2*-weighted angiography (ESWAN) in magnetic resonance imaging (MRI) to distinguish between cervical adenocarcinoma (CA) and cervical squamous carcinoma (CSC).

**Methods:**

Seventy-eight patients who underwent ESWAN from 2014 to 2019 were stratified into two groups: CA (26 patients) and CSC (52 patients). R2* values of the lesions were measured, and ITSS ratios were automatically calculated using the Anatomy Sketch (AS) software. Independent samples t-tests or Mann-Whitney U-tests were utilized to evaluate disparities in the parameters. Binary logistic regression was conducted to identify independent predictors. The receiver operating characteristic curve was employed to assess diagnostic value, and the Delong test was applied to compare differences in the area under the curve (AUC).

**Results:**

The CA group exhibited significantly higher values for the ITSSs, ITSSv and R2* value, lower alpha fetoprotein (AFP) and prognostic nutritional index (PNI) (ITSSs: 0.203 ± 0.111; ITSSv:0.206 ± 0.098; R2* value:20.340 ± 5.572Hz; AFP: 1.73(1.33,2.99)ng/ml; PNI:49.150(45.825,51.775)) than that of the CSC group (ITSSs: 0.072 ± 0.019; ITSSv: 0.076 ± 0.030; R2* value: 13.233 ± 4.083Hz; AFP: 2.99(1.88,2.99)ng/ml; PNI: 50.775(48.563,54.050)) (*P*< 0.05). Among them, ITSSv and R2* value were independent risk predictors. The AUC values for ITSSv, R_2_^*^ value and the combined model for differentiate between CA and CSC were 0.942, 0.851 and 0.950, respectively. The results of the Delong test indicated that the combined model exhibited superior diagnostic efficacy compared to R_2_^*^ value (*P*< 0.05), but no significant difference from ITSSv (*P*>0.05).

**Conclusion:**

ITSSv and R2* values derived from ESWAN facilitate the quantitative differentiate between CA and CSC. The automated extraction of ITSSv is convenient and reliable, making it a promising candidate for clinical implementation.

## Introduction

Cervical cancer (CC) is the fourth most common cancer among women worldwide and ranks as the second leading cause of cancer-related deaths in women aged 20–39 years in the United States (1, 2). It is also the fifth most prevalent cancer in China (3). Among the various types of CC, CSC and CA are the most prevalent, with CA accounting for approximately 20% of CC cases (4). In contrast to CSC, CA primarily originates from the cervical stroma, is more likely to infiltrate the lymphatic vascular space, and is associated with lymph node and distant metastases, resulting in a relatively aggressive clinical course, decreased sensitivity to radiotherapy (5) and a poor prognosis (6–8). Research indicates that the 5-year survival rate for CA is approximately 10%-20% lower than that for CSC (9). The stagnant survival trends for CC likely reflect, in part, an increased proportion of CA, making the identification of the two types of cancer significant. However, conventional multiple punch biopsy performed under vaginoscopy can be influenced by factors such as lesion size, sampling precision and the experience of operators (10), which may lead to variations in final pathological outcomes. Research indicates that around 24.3% of patients diagnosed with invasive CA exhibit negative cytological results (11). Given the notable differences in surgical treatment approaches and postoperative adjuvant chemotherapy between CA and CSC, a reliable method to accurately distinguish between the two prior to surgery is essential for enhancing patient survival rates (12).

In October 2018, the International Federation of Gynecology and Obstetrics (FIGO) updated the CC staging system and for the first time proposed that imaging findings could be utilized for CC staging (13), aiming to establish a comprehensive clinical/pathological/radiological staging framework, thereby imposing higher requirements for imaging examinations. ESWAN sequence, derived from susceptibility-weighted imaging, integrates techniques that leverage magnetic susceptibility differences and oxygen level-dependent effects. This approach facilitates the collection of quantitative parameters, such as transverse relaxation rate (R2*) values, through post-processing, enabling the non-invasive assessment of oxygenation and local metabolic status in hypoxic tumor regions (14, 15). In addition to visualizing and delineating small vessels and microbleeds, ESWAN also contributes to the observation of both physiological and pathological conditions (16).

Studies have demonstrated that intratumoral hemorrhages are frequently in the form of microhemorrhages (17). The ITSS appears as a low-signal region on the phase map, characterized by a pattern of continuous dots or slender lines within the tumor, primarily resulting from microhemorrhages and neovascularization (18). This feature serves as an intuitive and non-invasive imaging biomarker for assessing vascular proliferation in pathological tissues, effectively illustrating the density and dimensions of micro-vessels present in the lesion (19).

In this study, CA and CSC were identified through automatic ITSS extraction combined with R2* values derived from ESWAN, providing a non-invasive approach for predicting the pathological classification of CC.

## Materials and methods

### Patients and data collection

This retrospective study received approval from the Ethics Committee. The clinical and imaging data of CC patients who underwent 1.5T MRI at our hospital from April 2014 to December 2019 were retrospectively analyzed.

The inclusion criteria for the study were as follows: (1) patients with histologically confirmed CSC/CA following surgical resection; (2) high-quality MRI images, including T1-weighted imaging (T_1_WI), T2-weighted imaging (T_2_WI), diffusion-weighted imaging (DWI), and ESWAN sequences, which provided clear visualization of lesions, were free from artifacts, and facilitated the identification of tumor boundaries for effective region of interest (ROI) delineation.

Patients were excluded from the study for the following reasons: (1) the presence of other coexisting uterine diseases that could affect the observation and measurement of cervical lesions; (2) patients who had undergone any prior treatments, such as biopsy, curettage, radiotherapy, chemotherapy, or immunotherapy, before the MRI examination; (3) lesions measuring less than 1.0 cm or those for which the delineation process indicated fewer than three layers.

**Figure 1:**
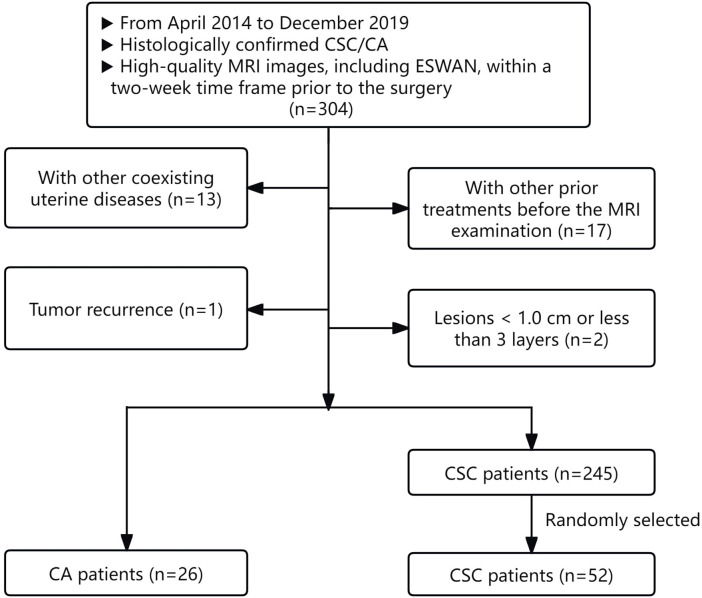
Flowchart of patient selection process.

According to the above criteria, 271 patients were enrolled, comprising 26 CA patients and 245 CSC patients. For the subsequent statistical analysis, 26 CA patients and 52 controls with CSC in a 1:2 ratio (randomly selected from 245 CSC patients) were ultimately included in our study (20). The flowchart of patient selection process was illustrated in **Figure 1**. The demographic and clinicopathological characteristics of the two groups were gathered from the hospital information system, including variables such as age, irregular vaginal bleeding, tumor size, FIGO stage, carcinoembryonic antigen, AFP, carbohydrate antigen 12-5, carbohydrate antigen 19-9, epididymal protein 4, neutrophil-lymphocyte ratio, monocyte-lymphocyte ratio, platelet-lymphocyte ratio, systematic immune inflammation index, pan-immune inflammation value, PNI, aspartate aminotransferase to lymphocyte ratio index, aspartate aminotransferase to platelet ratio index, degree of differentiation, lymphatic vascular space invasion, perineural invasion, and lymph node metastasis.

### MRI data acquisition

All patients enrolled in the study underwent Signa HDxt 1.5T MRI (GE Healthcare, Milwaukee, WI, USA) within a two-week time frame prior to surgery. Birth control rings were removed one day before the examination, and dietary abstinence was enforced for 4 to 6 hours to mitigate gastrointestinal peristaltic artifacts. Patients were instructed to consume approximately 500 ml of water one hour before the examination to achieve moderate bladder fullness, and they received guidance on proper breathing techniques to minimize respiratory motion artifacts that could compromise image quality. Patients were positioned supine with their feet advanced for the duration of the examination. The study employed an 8-channel phased array body coil for imaging, utilizing scanning sequences including T_1_WI, T_2_WI, DWI, dynamic contrast-enhanced MRI (DCE-MRI) and ESWAN. Detailed scanning parameters were provided in **Table 1**.

**Table 1:** Scanning sequence parameters.

Sequence	orientation	TR (ms)	TE (ms)	ACQ Voxel (mm^2^)	NEX	FOV (mm^2^)	Thickness/ Gap(mm)	Scan time
T_1_WI	AXI	680	10	320*192	2.0	30*30	1.0/1.0	1min 37s
T_2_WI	AXI	5660	88.4	288*224	3.0	30*30	5.0/1.0	3min 13s
DWI	AXI	3725	71.1	128*128	6.0	30*30	5.0/1.0	1min 15s
T_2_WI	SAG	3980	91.8	256*224	3.0	30*30	5.0/1.0	2min 55s
DWI	SAG	3725	71.1	192*192	6.0	31*31	5.0/1.0	1min 15s
LAVA	AXI	3.9	1.9	128*128	0.71	39*39	5.0/2.5	3min 29s
ESWAN	AXI	16.5	2.1/5.1/8.0/10.9/13.8	256*192	0.71	40*40	5.0/2.0	21 s

The 2 b values of DWI were 0, 1000 s/mm^2^. AXI, axial; DWI,diffusion-weighted imaging; ESWAN,enhanced T2*-weighted angiography; FOV, field of view; LAVA, liver acceleration volume acquisition; NEX, number of excitations; SAG, sagittal; TE, echo time; TR, repetition time; T_1_WI, T1-weighted imaging; T_2_WI, T2-weighted imaging.

### Measurement of ITSS ratios

The initial axial digital images obtained from the ESWAN sequence were transmitted to the Advantage Workstation (AW, Version 4.6; GE Healthcare, Milwaukee, WI, USA). Post-processing was conducted using the Functool software to generate the phase maps. Prior to measurement, an image de-artifacting program developed in Python was employed to eliminate artifacts from the phase maps. (1) Input a phase map requiring artifact removal, containing a band-like artifact characterized by alternating dark and bright pixel bands; pixels exhibiting these characteristics necessitate elimination. (2) Apply median filtering to the image in step 1 to generate a smoothed image. (3) Subtract the pixel values of the image in step 1 from the image in step 2. Pixels in the resulting matrix that fall below -150 are classified as having insufficient signal, while pixels exceeding 200 are deemed to have excessive signal. Assign -1 to pixels with insufficient signal, 1 to pixels with excessive signal, and 0 to the remaining pixels to create a new 3D array. (4) Extract the regions surrounding the values of 1 and -1 from the 3D array generated in step 3, expand the resulting area by 1 pixel in the cross-section, and designate the pixels within this region as pseudo-imaging pixels. (5) Recalculate the pixel values of the artifactual region by determining the average of the 26 neighboring non-artifactual region pixels for each target pixel, thereby replacing the original artifactual region pixel values. (6) Replace the corresponding pixel values in the original phase map from step 1 with the pixel values obtained in step 5, resulting in the generation of the resultant map. The Python-based artifact removal program can be found in the **Supplementary Material** named “Python-Based Artifact Removal Program.zip”.

Following the artifact removal process, the phase maps were exported to NII format using a batch program and subsequently transmitted to AS software, which incorporates C++ programming along with Qt and VTK libraries, for the calculation of ITSS ratios. The analysis codes are available in a GitHub repository (https://github.com/DlutMedimgGroup/AnatomySketch-Software).

**Figure 2:**
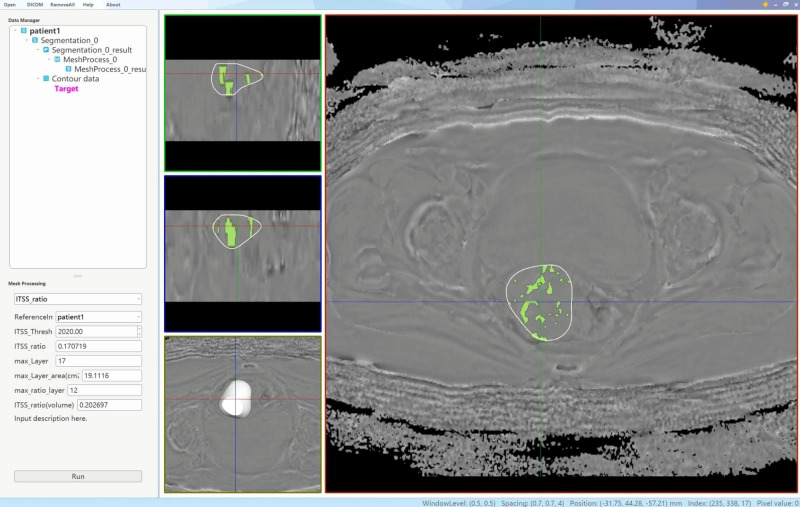
A schematic diagram of the AS software was utilized to calculate the tumor ITSS ratio. By employing the interpolation and labeling tools within the AS software, the ITSS ratio within the ROI was determined. The ITSS ratio is operationally defined as the ratio of pixels exhibiting low signal intensity within the ROI to the total number of pixels present within the ROI.

Two physicians, each with 6 and 11 years of experience in uterine MRI imaging diagnosis, utilized a double-blind method to identify tumor lesions. Referring to T_2_WI, DWI and apparent diffusion coefficient maps, the ROIs were delineated using an interactive semi-automatic method from the first to the last layer of the lesions on the phase maps. The edges of the lesions were defined based on DCE-MRI, with the ROIs outlined within 0.5 cm of the tumor edge to minimize volume effects. Therefore, lesions with a narrow range were excluded to ensure the accuracy of lesion delineation and the reliability of subsequent quantitative analysis. Upon completion of the delineations, three-dimensional volumes of interest were generated using the AS software. Subsequently, a plug-in within the AS software was utilized to determine the ITSS regions in the volumes of interest by establishing a threshold value of 2020. The ITSS ratios were then automatically calculated, including the ratio of the ITSS area in the maximum ITSS layer to the tumor area at that layer (intratumoral susceptibility signal sectional, ITSSs) and the ratio of tumor ITSS volume to the whole tumor volume (intratumoral susceptibility signal volume, ITSSv), as depicted in **Figure 2**.

### Measurement of R2* values of the ESWAN sequence

**Figure 3:**
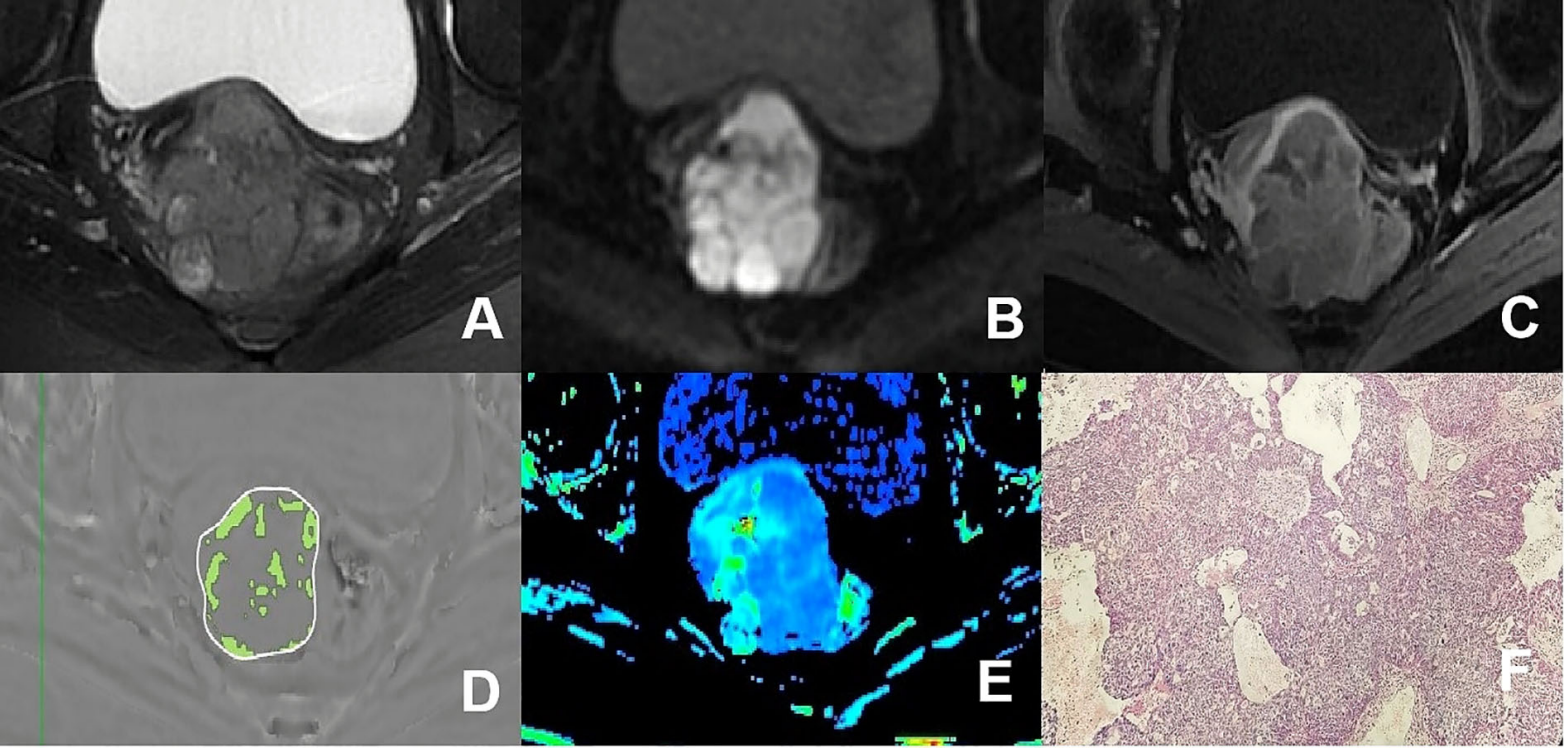
**(A–F)** presented a 75-year-old patient diagnosed with poorly differentiated CA, classified as FIGO stage IIB. **(A)** Axial T_2_WI image: the mass demonstrated mixed iso-hyperintense signal; **(B)** Axial DWI image: the mass with restricted diffusion showed significant hyperintense signal; **(C)** Axial enhanced image of the delayed period: the mass revealed uneven hypointense signal and annular enhancement; **(D)** The schematic diagram illustrated the ITSS intensity measured using AS software, where the green area on the diagram signified the ITSS within the tumor, with an ITSSs of 0.204 and ITSSv of 0.229; **(E)** R2* map of the ESWAN sequence, where the mean value of R2* for this patient was 23.184Hz. **(F)** Pathological image confirmed CA.

**Figure 4:**
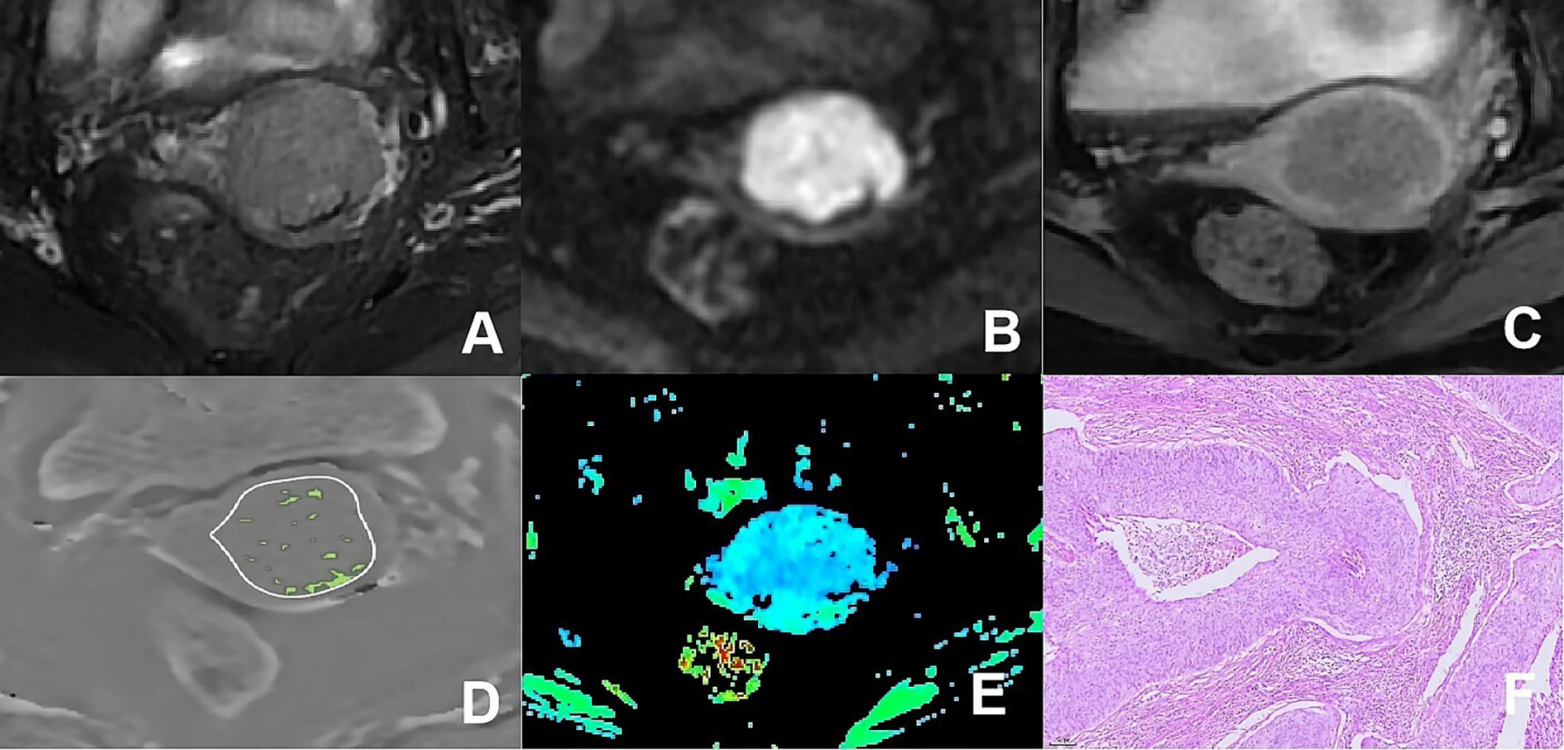
**(A–F)** presented a 66-year-old patient diagnosed with poorly differentiated CSC, classified as FIGO stage IIA. **(A)** Axial T_2_WI image: the mass presented isointense signal; **(B)** Axial DWI image: the mass with restricted diffusion exhibited significant hyperintense signal; **(C)** Axial enhanced image of the delayed period: the mass displayed low level of enhancement; **(D)** The schematic diagram illustrated the ITSS intensity measured using AS software, where the green area on the diagram signified the ITSS within the tumor, with an ITSSs of 0.091 and ITSSv of 0.077; **(E)** R2* map of the ESWAN sequence, where the mean value of R2* for this patient was 10.440Hz. **(F)** Pathological image confirmed CSC.

The ESWAN imaging post-processing was performed using the GE AW4.6 workstation equipped with Functool software to generate R2* maps. Without prior knowledge of the pathological results, two observers delineated irregularly shaped ROIs at the largest extent of the tumor parenchymal region, referencing T_2_WI, DWI, apparent diffusion coefficient maps and DCE-MRI. This process ensured that the ROIs encompassed the solid tumor area as comprehensively as possible while avoiding regions of necrosis, hemorrhage, and cystic degeneration. Measurements were taken three times, one month apart, to calculate an average. As illustrated in **Figures 3** and **4**.

### Statistical analysis

Statistical analysis was performed using SPSS 21.0 software (Chicago, IL, USA) and MedCalc 15.2.2 software (Med Calc Software, Ostend, Belgium). Intra-class correlation coefficients were utilized to evaluate the agreement between ITSSs, ITSSv, and R2* values measured by two observers. The Shapiro-Wilk test was employed to assess the normality of the measurements, and either the two independent samples t-test or the Mann-Whitney U-test was employed to compare quantitative parameters between CA and CSC groups. The clinicopathological data of the two groups were presented as frequencies or percentages, with group comparisons conducted using the chi-square test or Fisher’s exact test. Binary logistic regression was employed to identify independent predictors that differentiate CA from CSC. Receiver operating characteristic curve analysis was applied to assess the statistically significant parameters and their combinations for distinguishing between CA and CSC. The Delong test was utilized to compare the differences in the AUC.

## Results

### Comparison of the general clinicopathological data between the two groups

The AFP and PNI levels in the CA group were significantly lower than those in the CSC group (*P*< 0.05). Other general clinicopathological features, including age, irregular vaginal bleeding, tumor size, FIGO stage, carcinoembryonic antigen, carbohydrate antigen 12-5, carbohydrate antigen 19-9, epididymal protein 4, neutrophil-lymphocyte ratio, monocyte-lymphocyte ratio, platelet-lymphocyte ratio, systematic immune inflammation index, pan-immune inflammation value, aspartate aminotransferase to lymphocyte ratio index, aspartate aminotransferase to platelet ratio index, degree of differentiation, lymphatic vascular space invasion, perineural invasion and lymph node metastasis, were analyzed; however, the differences were not statistically significant (*P*>0.05). As shown in **Table 2**.

**Table 2:** Comparison of general clinical data of two groups of patients.

Characteristics	n	CSC(n=52)	CA(n=26)	χ^2^/Z	*P*
Age (year)	78	52.19±10.34	56.77±10.83	35.786	0.182*
Irregular vaginal bleeding n/%					
No	13	8/52 (15.38%)	5/26 (51.88%)	0.185	0.751^1^
Yes	65	44/52 (84.62%)	21/26 (48.12%)		
Tumor size (cm)	78	3.45(2.60,4.20)	3.30(2.08.5.00)	-0.286	0.775^#^
FIGO stage n/%					
I stage	47	35/52 (67.31%)	12/26 (46.15%)	4.965	0.081*
II stage	23	11/52 (21.15%)	12/26 (46.15%)		
III stage	8	6/52 (11.54%)	2/26 (7.69%)		
CEA (ng/ml)	78	1.61(1.07,2.30)	2.73(1.06,5.03)	-1.111	0.267^#^
AFP (ng/ml)	78	2.99(1.88,2.99)	1.73(1.33,2.99)	-2.901	***0.004^#^***
CA12-5 (U/ml)	78	19.43(10.09,67.91)	27.46(12.99,109.85)	-1.210	0.226^#^
CA19-9 (U/ml)	78	10.71(6.16,19.10)	17.17(9.43,28.08)	-1.767	0.077^#^
HE4 (pmol/L)	78	53.48(44.47,66.36)	64.25(48.14,90.81)	-1.062	0.109^#^
NLR	78	1.705(1.187,2.331)	1.951(11.325,2.640)	-1.518	0.129^#^
MLR	78	0.195(0.167,0.271)	0.219(0.151,0.341)	-0.490	0.624^#^
PLR	78	128.067(106.091,170.874)	143.429(101.381,173.622)	-0.305	0.761^#^
SII	78	414.388(276.503,637.761)	400.378(273.347,675.647)	-0.359	0.720^#^
PIV	78	161.840(96.449,235.306)	140.132(84.169,237.374)	-0.207	0.836^#^
PNI	78	50.775(48.563,54.050)	49.150(45.825,51.775)	-2.401	***0.016^#^***
ALRI	78	9.078(6.954,12.146)	10.048(7.199,17.037)	-1.425	0.154^#^
APRI	78	0.069(0.050,0.093)	0.078(0.056,0.114)	-1.001	0.317^#^
Degree of differentiation n/%					
Low	48	35/52 (67.31%)	13/26 (50.00%)	2.194	0.217^1^
Middle/High	30	17/52 (32.69%)	13/26 (50.00%)		
LVSI n/%					
No	49	29/52 (62.79%)	20/26 (65.41%)	3.321	0.085^1^
Yes	29	23/52 (37.21%)	6/26 (34.59%)		
Perineural invasion n/%					
No	71	46/52 (75.58%)	25/26 (85.71%)	1.256	0.414^1^
Yes	7	6/52 (24.42%)	1/26 (14.29%)		
LNM n/%					
No	70	46/52 (75.58%)	24/26 (92.48%)	0.279	0.712^1^
Yes	8	6/52 (24.42%)	2/26 (7.52%)		

The independent samples t-test was applied to the data marked "^1^"; Fisher's exact test was applied to the data marked "^*^"; and the Mann- Whitney U test was applied to the data marked "^#^". Data in bold are statistically significant. AFP, alpha-fetoprotein; ALRI, aspartate aminotransferase to lymphocyte ratio index; APRI, aspartate aminotransferase to platelet ratioindex; CA, cervical adenocarcinoma; CA12-5, carbohydrate antigen 12-5; CA19-9, carbohydrate antigen 19-9; CEA, carcinoembryonic antigen; CSC,cervical squamous carcinoma; FIGO, International Federation of Gynecology and Obstetrics; HE4, epididymal protein 4; LNM, lymph node metastasis; LVSI, lymphatic vascular space invasion; MLR, monocyte-lymphocyte ratio; NLR, neutrophil-lymphocyte ratio; PIV, pan-immune inflammation value; PLR, platelet-lymphocyte ratio; PNI, prognostic nutritional index; SII, systematic immune inflammation index.

### Consistency analysis of the measurements between the two observers

The inter-rater reliability of the ITSSs, ITSSv and R2* values, as assessed by the two observers, was deemed satisfactory, with all intra-class correlation coefficients values exceeding 0.75, as indicated in **Table 3**. The average of the measurements obtained from the two observers was utilized for subsequent analysis.

**Table 3:** Comparison of consistency of measurement data between two observers.

Parameters	Group	Observer1	Observer2	ICC(95%CI)
ITSSs(%)	CSC	0.073±0.019	0.071±0.021	0.944(0.905,0.968)
	CA	0.202±0.116	0.204±0.109	0.968(0.930,0.986)
ITSSv(%)	CSC	0.078±0.031	0.073±0.029	0.944(0.904,0.967)
	CA	0.211±0.100	0.201±0.099	0.963(0.918,0.984)
R_2_^*^ value (Hz)	CSC	13.134±4.232	13.326±4.243	0.923(0.866,0.956)
	CA	20.279±6.040	20.401±5.419	0.940(0.865,0.973)

CA, cervical adenocarcinoma; CSC, cervical squamous carcinoma; ICC, intra-class correlation coefficients; ITSSs, intratumoral susceptibility signal sectional; ITSSv, intratumoral susceptibility signal volume.

### Comparison of the ITSSs, ITSSv and R2* values between the two groups

**Figure 5:**
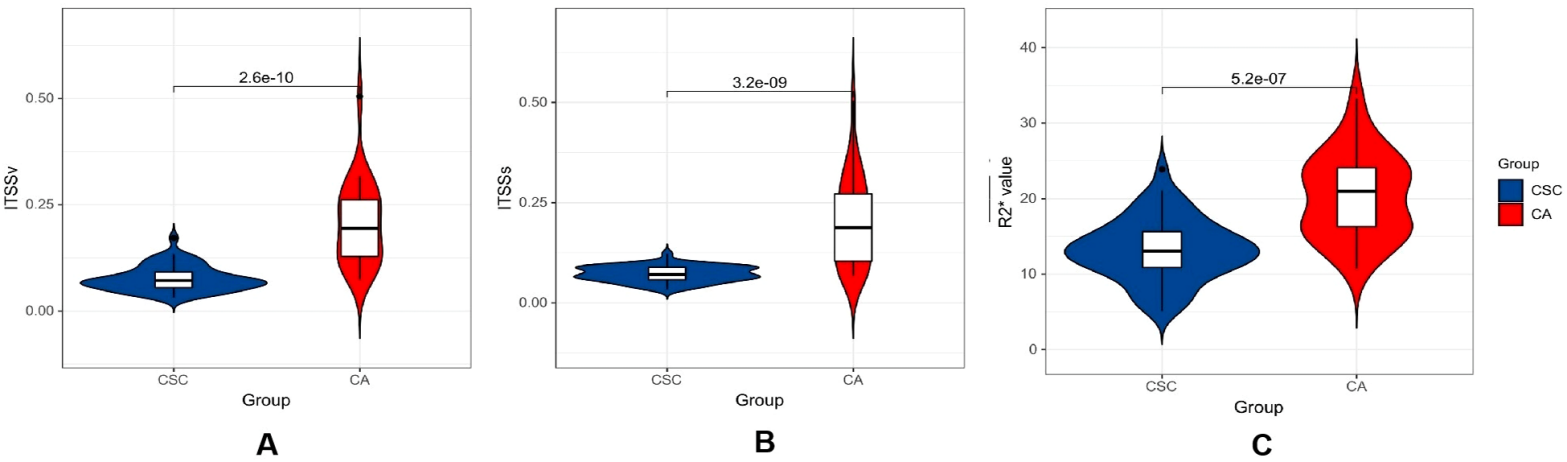
Violin plot displaying the distribution of parameters within CA and CSC group. **(A–C)** differential comparison of ITSSv, ITSSs and R2* value between two groups of patients.

The ITSSs, ITSSv and R2* values of the CA group were significantly higher than those of the CSC group (*P*< 0.05), as shown in **Table 4** and **Figure 5**.

**Table 4:** Comparison of the differences between the parameters in the two groups of patients.

Parameters	CSC(n=52)	CA(n=26)	t/z	*P*
ITSSs (%)	0.072±0.019	0.203±0.111	-5.925	***<0.001***
ITSSv (%)	0.076±0.030	0.206±0.098	-6.328	***<0.001***
R_2_^*^ value (Hz)	13.233±4.083	20.340±5.572	-5.024	***<0.001***

Data in bold were statistically significant. CA, cervical adenocarcinoma; CSC, cervical squamous carcinoma; ITSSs, intratumoral susceptibility signal sectional; ITSSv, intratumoral susceptibility signal volume.

### Independent predictors for differentiating between CA and CSC

The comprehensive clinicopathological data and various quantitative MRI parameters with *P* values below 0.1 between the two groups were incorporated into the subsequent analysis. Following univariate and multivariate logistic regression analyses, the ITSSv and R2* value emerged as independent predictors for differentiating between CA and CSC, as illustrated in **Table 5**.

**Table 5:** Univariate and multivariate analyses to distinguish CA and CSC.

Parameters	Univariate analysis		Multivariate analysis	
	OR (95%CI)	*P*	OR (95%CI)	*P*
FIGO stage	2.467 (0.930 - 6.541)	0.0700		
LVSI	0.378 (0.131 - 1.096)	0.0730		
AFP (ng/ml)	0.502 (0.287 - 0.876)	***0.0154***		
CA19-9 (U/ml)	1.000 (1.000 - 1.001)	0.4740		
PNI	0.866 (0.768 - 0.977)	***0.0193***		
ITSSs(%)	1.770 (1.235 - 2.536)	***<0.0001***		
ITSSv (%)	1.586 (1.257- 2.002)	***<0.0001***	1.475 (1.175 - 1.852)	***0.0008***
R_2_^*^ value (Hz)	1.371(1.185- 1.588)	***<0.0001***	1.198 (0.996 - 1.441)	***0.0555***

All variables with *P*< 0.1 in the univariate analysis were included in the multivariate regression analysis. Bold text in the table indicates statistically significant logistic regression analysis. AFP, alpha-fetoprotein; CA, cervical adenocarcinoma; CA19-9, carbohydrate antigen 19-9; CI, confidence interval; CSC, cervical squamous carcinoma; FIGO, International Federation of Gynecology and Obstetrics; ITSSs, intratumoral susceptibility signal sectional; ITSSv, intratumoral susceptibility signal volume; LVSI, lymphatic vascular space invasion; OR, odds ratio; PNI, prognostic nutritional index.

### Efficiency of ITSSv, R2* value and the combined model for differentiating between CA and CSC

**Figure 6:**
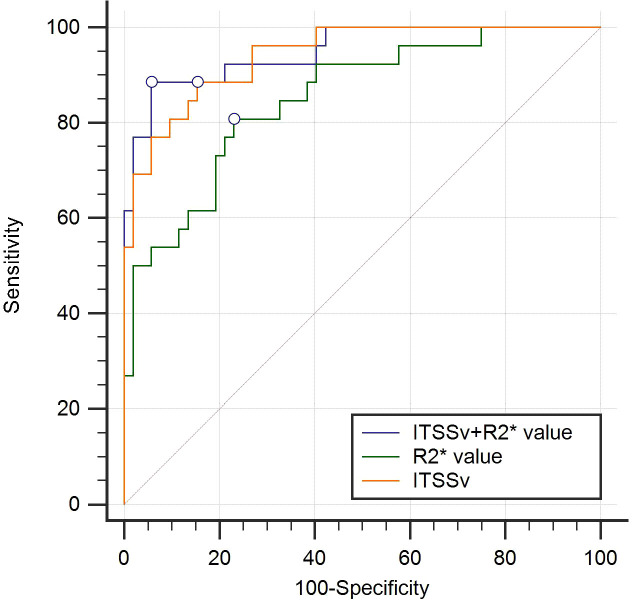
Receiver operating characteristic curves analyzing the efficacy of ITSSv+R2*value, R2* value and ITSSv to distinguish CA and CSC with AUC values of 0.950, 0.851 and 0.942, respectively.

The AUC for ITSSv, R2* value, and the combined model for differentiating between CA and CSC were 0.942, 0.851, and 0.950, respectively, as depicted in **Figure 6**. The Delong test indicated that the diagnostic efficacy of the combined model was superior to that of the R2* value (*P*< 0.05); however, there was no significant difference when compared to ITSSv (*P*>0.05), as shown in **Table 6**.

**Table 6:** The efficiency of ITSSv, R_2_^*^ value and the combined parameter to distinguish CA and CSC.

Parameters	AUC (95%CI)	Thresholds	Sensitivity (%)	Specificity (%)	Delong test	
ITSSv (%)	0.942 (0.864 - 0.982)	0.107	88.46	84.62	Z=0.691	*P*=0.4898
R_2_^*^ value (Hz)	0.851 (0.752 - 0.921)	15.698	80.77	76.92	Z=2.858	***P=0.0043***
ITSSv+R_2_* value	0.950(0.876 - 0.987)	0.264	88.46	94.23	NA	NA

Data in bold are statistically significant. AUC, area under the curve; CA, cervical adenocarcinoma; CI, confidence interval; CSC, cervical squamous carcinoma; ITSSv, intratumoral susceptibility signal volume.

Pairwise comparisons of the single parameters ITSSs, ITSSv, R2* value, and the combined models were compared pairwise. The Delong test revealed that the AUCs for ITSSv compared to R2* value, ITSSs + R2* value compared to R2* value, ITSSv + R2* value compared to R2* value, and ITSSs + ITSSv + R2* value compared to R2* value between the two groups were statistically significant (*P*< 0.05), as presented in **Table 7**.

**Table 7:** Comparison of multiple quantitative parameters and combined parameters to distinguish CA and CSC.

Parameters	AUC difference value(95%CI)	Delong test	
ITSSv (%) - ITSSs (%)	0.029(0.036-0.017)	Z=1.203	*P*=0.2291
ITSSv (%) - R_2_^*^ value (Hz)	0.091(0.112 - 0,061)	Z=2.137	***P=0.0326***
ITSSs (%) - R_2_^*^ value (Hz)	0.062(0.076 - 0.044)	Z=1.357	*P*=0.1749
ITSSs+R_2_* value - R_2_^*^ value (Hz)	0.086(0.106 - 0.059)	Z=2.473	***P=0.0134***
ITSSs+R_2_* value - ITSSs (%)	0.024(0.030 - 0.015)	Z=1.344	*P*=0.1790
ITSSv (%) - ITSSs+R_2_* value	0.005(0.006 - 0.002)	Z=0.221	*P*=0.8250
ITSSv+R_2_* value - R_2_^*^ value (Hz)	0.099(0.124 - 0.066)	Z=2.858	***P=0.0043***
ITSSv+R_2_* value - ITSSs (%)	0.037(0.048 - 0.022)	Z=1.484	*P*=0.1377
ITSSv+R_2_* value - ITSSv (%)	0.008(0.012 - 0.005)	Z=0.691	*P*=0.4898
ITSSv+R_2_* value - ITSSs+R_2_* value	0.013(0.018 - 0.007)	Z=0.963	*P*=0.3357
ITSSs+ITSSv+R_2_* value - R_2_^*^ value (Hz)	0.099(0.124 - 0.066)	Z=2.858	***P=0.0043***
ITSSs+ITSSv+R_2_* value - ITSSs (%)	0.037(0.048 - 0.022)	Z=1.484	*P*=0.1377
ITSSs+ITSSv+R_2_* value - ITSSv (%)	0.008(0.012 - 0.005)	Z=0.691	*P*=0.4898
ITSSs+ITSSv+R_2_* value - ITSSs+R_2_* value	0.013(0.018- 0.007)	Z=0.963	*P*=0.3357

Data in bold were statistically significant. AUC, area under the curve; CA, cervical adenocarcinoma; CI, confidence interval; CSC, cervical squamous carcinoma; ITSSs, intratumoral susceptibility signal sectional; ITSSv, intratumoral susceptibility signal volume.

## Discussion

The primary discovery of this study was the efficacy of automated ITSS intensity extraction in conjunction with R2* values for differentiating between CA and CSC. Our research revealed that the ITSSs, ITSSv and R2* values in the CA group were significantly higher than those in the CSC group. Furthermore, after controlling for confounding variables, ITSSv and R2* values emerged as independent factors. The combined model demonstrated high diagnostic efficacy in distinguishing between CA and CSC, revealing significant differences when compared to R2* values alone.

In this study, the clinical manifestations of patients with CA and CSC were found to be similar, primarily, presenting as irregular vaginal bleeding. There was no statistically significant difference between the two groups, which complicates differentiation. Most laboratory tests indicated no obvious discrepancies between CA and CSC; however, AFP and PNI exhibited significant differences. AFP, a glycoprotein belonging to the serum albumin gene family, is commonly utilized as a biomarker for liver cancer. Nevertheless, He et al. (21) observed elevated serum AFP levels in patients with various cancers and non-cancerous diseases, including CC. The results of this study demonstrated that AFP levels in the CA group were significantly lower than those in the CSC group, suggesting its potential clinical utility, although the underlying molecular mechanisms warrant further investigation. PNI, derived from serum albumin levels and peripheral blood lymphocyte counts, serves as an important indicator of both nutritional status and systemic inflammation. Serum albumin, an acute-phase protein, is linked to systemic inflammation, while lymphocytes are essential components of cell-mediated immunity. Both nutrition and inflammation significantly influence tumorigenesis, cancer progression, and metastasis by affecting the tumor microenvironment (22). Therefore, PNI may serve as a powerful prognostic indicator for patients with various types of cancer, as confirmed by numerous studies (23–27). Niu et al. (28) conducted a meta-analysis that revealed a significant association between decreased PNI and poorer overall survival and progression-free survival, as well as an increased likelihood of tumor metastasis and higher tumor burden. The results of this study indicated that PNI values in the CA group were notably lower than those in the CSC group, suggesting that CA represents a more advanced tumor with a worse prognosis compared to CSC. However, the diagnostic efficacy of AFP and PNI was found to be limited, with AUC of 0.698 and 0.667, respectively. Consequently, this study aims to explore the value of imaging indicators in the differential diagnosis of CA and CSC.

Currently, the predominant assessments of ITSS primarily rely on semi-quantitative methods (17, 29–36). Bhattacharjee et al. (37) attempted to conduct a quantitative analysis of ITSS in brain glioma. However, these approaches are constrained by several limitations. Firstly, manual counts in previous studies have not always been consistent, likely due to the absence of an established objective standard for ITSS quantification. This inconsistency is further exacerbated by the challenges associated with manually calculating ITSS, which is susceptible to subjective interpretation by the evaluator, resulting in inadequate reproducibility. Secondly, the focus on the head as the study subject, which experiences minimal interference from respiratory and motion artifacts, highlights the need for further investigation into the applicability and reliability of these methods for abdominal organs. Thirdly, the failure to address phase diagram artifacts may result in inaccuracies in the findings. To overcome the limitations of existing methodologies, this study utilized the ESWAN sequence for pre-processing phase map artifacts, specifically targeting challenges related to respiratory and motion-induced artifacts in abdominal organs. Furthermore, an automated approach was implemented to extract the ITSS ratios for patients with CC, calculated based on the ratio of low-signal region pixels within the tumor to the total number of pixels in both the maximum ITSS layer (ITSSs) and the whole tumor volume (ITSSv). This method is characterized by its simplicity, reproducibility, and reduced subjectivity, rendering it highly suitable for clinical applications.

In this study, we demonstrated the potential utility of ITSS in differentiating between CA and CSC. ITSS provides a comprehensive depiction of neovascularization and microhemorrhage within tumors, serving as a noninvasive and intuitive imaging marker for vascular proliferation. Our findings indicated that ITSSs and ITSSv were significantly higher in the CA group compared to the CSC group. This observation can be attributed to the more malignant and aggressive nature of CA, which expresses markedly higher levels of vascular endothelial growth factor-A than CSC (38). This over expression leads to neovascularization characterized by thin walls, high vascular permeability, and frequent microhemorrhages, thereby contributing to the elevated ITSSs and ITSSv. Furthermore, ITSS ratios offer the advantage of automated quantification, simplifying implementation and minimizing subjective bias when compared to other quantitative parameters, such as R2*, derived from ESWAN.

ESWAN is a type of magnetic-sensitive sequence, specifically a multi-echo T2*-weighted 3D gradient echo sequence, that reflects the magnetic-sensitive characteristics of tissues (39). The R2* value represents the apparent transverse relaxation rate, which is obtained through gradient echo imaging at different time intervals. The presence of paramagnetic substances, such as iron compounds, can induce inhomogeneity in the magnetic field, resulting in accelerated proton phase dispersion. This phenomenon leads to a more rapid attenuation of the transverse magnetization vector and an increase in the R2* value. Consequently, the R2* value is directly related to the concentration of deoxyhemoglobin containing iron ions. A higher R2* value, indicative of increased deoxyhemoglobin, suggests lower oxygen content, thereby allowing the R2* value to quantitatively evaluate changes in local tissue oxygen levels (40). Typically, paramagnetic substances, including iron and hemosiderin, influence the R2* value. Notably, the liver, which is the primary organ in the body that stores iron, exhibits characteristics that differ from those of other tissues or organs; however, to date, no evidence of iron deposition has been found in normal cervical tissue or cervical lesions (41, 42). Therefore, we conclude that R2* has promising applications in the detection of hypoxia in cervical cancer (43).

Currently, the R2* value has been preliminarily applied in the context of diseases affecting the female pelvic system (15, 44). However, its application in CC is limited and warrants further exploration. Li et al. (45) investigated the R2* value as a predictor of prognosis in advanced CSC treated with concurrent chemoradiotherapy. Additionally, research by Li-Ou Z et al. (43) confirmed a moderate correlation between activated hypoxia inducible factor-1α expression and the R2* value in CC. Our study found that R2* values were significantly higher in the CA group compared to the CSC group. CA is characterized by relatively aggressive tumor behavior (46), with tumor cells exhibiting rapid proliferation and metabolism. This results in an increased uptake of nutrients, including sugars and proteins, along with heightened oxygen consumption (47). Hypoxia prompts tumor cells to release various angiogenic factors, stimulating the formation of immature neovascularization, which further reduces oxygen saturation. Consequently, the increase in deoxygenated hemoglobin leads to elevated R2* values, resulting in higher R2* values in the CA group compared to the CSC group.

In this study, ITSSv and R2* values were identified as independent factors for distinguishing between CA and CSC. The combined model utilizing ITSSv and R2* values demonstrated the highest diagnostic efficiency, achieving an AUC of 0.950; notably, the inclusion or exclusion of ITSSs did not influence the results. The combined model outperformed the R2* value, but did not surpass ITSSv, indicating that ITSSv carries greater significance within the model compared to both ITSSs and R2* values. This can be attributed to the following reasons: (1) The comprehensive three-dimensional evaluation provided by ITSSv offers a holistic view of the lesions, particularly for irregular and complex formations. In contrast, ITSSs only capture the maximum ITSS layer of the lesion, which fails to convey complete information. (2) ITSSv is derived from the automatic extraction of the AS software and is calculated through volume delineation, streamlining the process and effectively minimizing variability between observers. Conversely, the R2* value can be influenced by subjective interpretations among observers regarding the solid tumor portions and lesion boundaries, potentially affecting the accuracy of the results. Furthermore, utilizing a ratio to characterize ITSS intensity provides a more accurate reflection of the relationship between the ITSS components and the entire tumor. This method also enables more effective comparisons between lesions, which is preferable to the ITSS volume value employed by Bhattacharjee et al. (36).

The limitations of this study are as follows: (1) The limited number of cases of CA restricted the ability to refine the pathological classification of CA, including subtypes such as mucinous adenocarcinoma and gastric-type adenocarcinoma. (2) This study was conducted at a single center and involved a relatively small sample size. Future research will aim to increase the sample size and conduct a multi-center study to facilitate a more comprehensive analysis.

## Conclusion

In conclusion, the combination of ITSSv and R2* values derived from ESWAN has the potential to significantly enhance the accuracy of differentiating between CA and CSC. By integrating the automated extraction process of ITSS with AS software, both two-dimensional (ITSSs) and three-dimensional (ITSSv) parameters can be obtained concurrently. ITSSv serves as an independent risk predictor, demonstrating notable advantages over ITSSs and exhibiting strong diagnostic efficacy comparable to the combined use of ITSSv and R2* values. This approach facilitates a comprehensive assessment of lesions and yields more precise information. This advancement is poised to supplant traditional R2* value measurements, presenting promising prospects for future clinical applications and widespread adoption.

## Data Availability

The data generated and analyzed during this study cannot be made publicly available due to regulations at the institutional review board concerning the potential of disclosure of an individual’s personal health information. Please contact the corresponding author regarding access to anonymized data.
